# Malignancies after vascularized composite allotransplantation

**DOI:** 10.3389/fimmu.2026.1905254

**Published:** 2026-07-28

**Authors:** Thomas Schaschinger, Tobias Niederegger, David Doll, Robert Munzinger, Gabriel Hundeshagen, Max Heiland, Curtis L. Cetrulo, Alexandre G. Lellouch, Leonard Knoedler

**Affiliations:** 1 University of Heidelberg, Medical Faculty Heidelberg, Heidelberg, Germany; 2 Department of Hand, Plastic and Reconstructive Surgery, Burn Center, BG Trauma Hospital Ludwigshafen, University of Heidelberg, Ludwigshafen, Germany; 3 Department of Plastic and Hand Surgery, Burn Center, BG Trauma Hospital Ludwigshafen, University of Heidelberg, Ludwigshafen, Germany; 4 Department of Oral and Maxillofacial Surgery, Charité–Universitätsmedizin Berlin, corporate member of Freie Universität Berlin and Humboldt-Universität zu Berlin, Berlin, Germany; 5 Division of Plastic and Reconstructive Surgery, Cedars-Sinai Medical Center, Los Angeles, CA, United States; 6 Vascularized Composite Allotransplantation Laboratory, Massachusetts General Hospital, Harvard Medical School, Boston, MA, United States; 7 Innovative Therapies in Haemostasis, INSERM UMR-S 1140, University of Paris, Paris, France

**Keywords:** malignancy, skin cancer, systematic review, transplantation, vascularized composite allotransplantation

## Abstract

**Background:**

Vascularized composite allotransplantation (VCA) has emerged as a reconstructive option for complex tissue defects, yet its oncologic long-term safety remains poorly characterized.

**Aims:**

This study aims to provide the most comprehensive overview of post-VCA malignancies to date.

**Methods and results:**

A systematic review was conducted following Preferred Reporting Items for Systematic Reviews and Meta-Analyses (PRISMA) guidelines, searching PubMed/MEDLINE, EMBASE, and Web of Science for malignancies that emerged after VCA. Additionally, registry cases were identified through the Organ Procurement and Transplantation Network (OPTN). Risk of bias and methodological quality of included case reports were assessed using the Joanna Briggs Institute (JBI) Critical Appraisal Checklist for Case Reports and Case Series. The review protocol is registered on PROSPERO (CRD420261350024). 13 articles met the inclusion criteria, comprising 13 patients with 18 malignancies, reflecting multiple malignancies in individual patients. A total of 15 patients with 22 malignancies, including two additional OPTN registry cases, were analyzed. The most frequently diagnosed malignancies included non-melanoma skin cancer (NMSC; 41%; n=9/22 malignancies) and lymphoproliferative disorders (36%; n=8/22 malignancies). One malignancy was a recurrence in a patient who was transplanted due to squamous cell carcinoma of the mouth. Median time to malignancy was 24 (interquartile range [IQR]: 12-72) months after transplantation, malignancy-associated mortality was 33% (n=5/15 patients) and mean survival time after VCA was 74 ± 58 months in these patients. Notably, no malignancy was identified at the graft site and 23% (n=5/22) of malignancies led to a modification of the immunosuppressive regimen. Two patients self-reported symptoms prior to their scheduled follow-up, including cutaneous morphologic changes and systemic symptoms (night sweats, dyspnea, swelling).

**Conclusion:**

In our cohort, post-VCA malignancies were associated with a malignancy-associated mortality of 33%, with NMSC (41%) and lymphoproliferative disorders (36%) predominating the spectrum. Notably, two patients self-reported symptoms prior to their scheduled follow-up, suggesting that structured patient education may be warranted. Given the elective nature of VCA, careful patient selection, structured dermatologic screening, and Epstein-Barr virus surveillance may be useful to enable early detection of post-VCA malignancies.

**Systematic review registration:**

https://www.crd.york.ac.uk/prospero/, identifier CRD420261350024.

## Graphical Abstract

**Figure:**
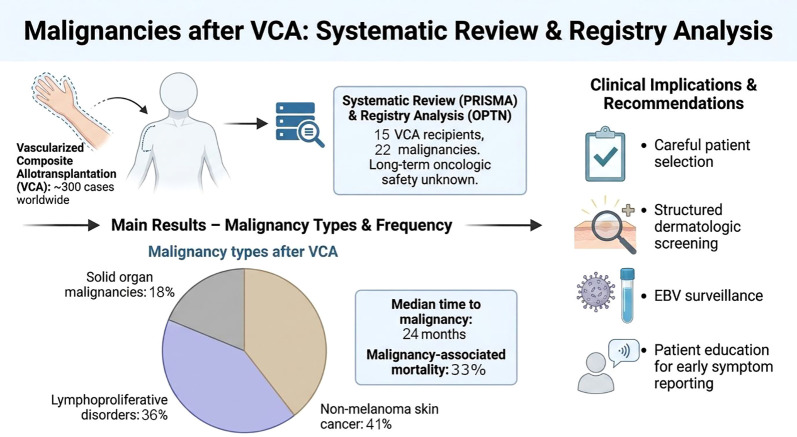

## 1 Introduction

Vascularized composite allotransplantation (VCA) has transformed reconstructive surgery since the first hand VCA in 1998 (1). To date, more than 300 VCAs have been performed worldwide (2–8). The indications and procedural complexity have steadily expanded, and long-term survival with functioning grafts is increasingly reported (9, 10). In this setting, oncologic safety is a central concern: VCA is not lifesaving, but requires lifelong immunosuppression, exposing predominantly young recipients to cumulative risks of infection, metabolic disease, and postoperative malignancies (11–14). Should malignancies occur, they may necessitate modification of immunosuppression and can result in graft loss, treatment-related morbidity or patient death (15–18).

In SOT (solid organ transplant) recipients, non-melanoma skin cancer (NMSC) and post-transplant lymphoproliferative disorder (PTLD) are among the most frequently reported malignancies and a leading cause of post-transplant mortality, with postoperative risk of squamous cell carcinoma (SCC) increasing up to 100-fold (19–21). They have been characterized in large national registries and population-based cohorts (20–22). By contrast, current knowledge on post-VCA malignancies relies on scattered case reports and small patient series (9, 11, 23).

However, the relative risk and spectrum of malignancies following VCA have not been systematically characterized and compared to those observed in SOT recipients (8, 11). To address this knowledge gap, we conducted this systematic review and supplemented it with the post-VCA malignancy cases that were identifiable in the OPTN registry under our search criteria, in order to provide the most comprehensive overview of post-VCA malignancies to date.

This review aims to support clinicians in post-transplant follow-up after VCA, with a particular emphasis on early malignancy detection, dermatologic screening and pre-operative patient selection. While the available evidence does not allow precise risk quantification, it may inform patient counseling regarding long-term oncologic risk. As VCA is performed electively and requires lifelong immunosuppression, these findings may directly guide surveillance protocols and help identify higher-risk candidates in whom conventional reconstruction may be preferable.

## 2 Methods

This systematic review of case reports and case series was conducted in accordance with the Preferred Reporting Items for Systematic Reviews and Meta-Analyses (PRISMA) guidelines. Given the nature of individual patient reports, we opted for a narrative synthesis. The review protocol is registered on PROSPERO (CRD420261350024).

In addition to the reported literature, a search of the OPTN (Organ Procurement & Transplantation Network) registry identified three post-VCA malignancy cases. The OPTN is a United States-based national registry administered by the United Network for Organ Sharing (UNOS), to which all federally designated US transplant centers are legally required to report transplant procedures and recipient outcomes. Since 2014, VCA has been classified as a transplantable tissue type within the OPTN framework, and as this reporting obligation is restricted to the United States, the OPTN does not capture VCA recipients treated at non-US institutions (24). These registry-derived cases were incorporated into the combined analysis presented in **Tables 1**, **2** alongside cases identified through the systematic literature search. To identify potential overlap between registry-derived and literature-derived cases, records were cross-checked for concordance across VCA-type, transplant timing, time-to-diagnosis, diagnostic modality, and immunosuppressive regimen.

**Table 1:** Summary of clinical and oncologic characteristics of the combined cohort.

Author	Type of malignancy	Type of VCA	Date of transplantation	Age [years]	Gender	Time of diagnosis [months]	Date of diagnosis	Modality of diagnosis	Treatment	Follow-up [months]	Outcome of malignancy
Zaccardelli et al.	PTLD	Bilateral upper e.	2011-10	65	m	125	2022-04	Clinical	Immuno	137	Remission
Conrad et al. ^a^	Lymphoma	Face	2009-11	27	m	5	2010-04	n/a	Immuno	36	Remission
Conrad et al. ^a^	EBV-PTSMT	Face	2009-11	27	m	24	2011-11	Imaging	Chemo	36	Stable
Landin et al.	BCC	Bilateral upper e.	2006	48	f	12	2007	Clinical	Surgery	12	Remission
Özkan et al. ^b^	PTLD	Face	2013-08-23	54	m	6	2014-02	n/a	n/a	11	Death
Özkan et al. ^b^	SCC	Face	2013-08-23	54	m	5	2014-01	Clinical	Surgery	11	Remission
Cavadas et al.	PTLD	Bilateral lower e.	2011-07	22	m	15	2012-12	Clinical	Chemo + Radio	38	Remission
Jozaghi et al.	BCC	Combined VCA and SOT	2015-05	55	m	12	2016-05	Clinical	Surgery	60	Remission
Kanitakis et al. ^c^	BCC	Face	2005-11-27	38	f	72	~2011-11 (6 years post-VCA)	Clinical	Surgery	125	Remission
Morelon et al. ^c^	SCLC	Face	2005-11-27	38	f	121	2015-12	Imaging	Surgery	125	Death
Kanitakis et al. ^c^	HPV+ CIS	Face	2005-11-27	38	f	48	~2009-11 (4 years post-VCA)	n/a	Surgery	125	Remission
Kermer et al.	SCC	Tongue	n/a*	42	m	12	12 months post-VCA	Clinical	No treatment	13	Progressive disease
Hautz et al.	GC	Unilateral upper e.	2009-07-22	55	m	96	2017 (8 years post-VCA)	n/a	Chemo	96	Death
Hautz et al. ^d^	BCC	Bilateral upper e.	2000-03-07	47	m	n/a	n/a	n/a	n/a	228	Remission
Hautz et al. ^d^	SCC	Bilateral upper e.	2000-03-07	47	m	n/a	n/a	n/a	n/a	228	Remission
Kollar et al.	HCC	Face	2009-04	59	m	65	2014-09	Imaging	Local ablation	121	Death
Cipriani et al.	PTLD	Abdominal Wall	2005-02	41	m	15	~2006-05 (15 months post-VCA)	n/a	n/a	15	Death
Kaufman et al.	PTLD	Unilateral upper e.	2006	n/a	m	22	2008 (22 m. post-VCA)	Blood draw	n/a	36	Stable
Registry case 1	PTLD	Unilateral upper e.	2006-11-29	54	m	22	2008-10-03	n/a	n/a	23	n/a
Registry case 2	SCC	Unilateral upper e.	2012-02-15	56	m	74	2018-04-14	n/a	n/a	74	n/a
Registry case 2	BCC	Unilateral upper e.	2012-02-15	56	m	50	2016-04-05	n/a	n/a	74	n/a
Registry case 2	Prostate Cancer	Unilateral upper e.	2012-02-15	56	m	64	2017-06-09	n/a	n/a	74	n/a
Registry case 3	PTLD	n/a	2019-02-25	31	f	n/a	n/a	n/a	n/a	7	n/a

*Exact date of transplantation not reported in the original publication; only relative postoperative timepoints (months) were provided, without anchoring calendar date. The manuscript was published February 27, 2008.

Identical patients with multiple malignancies are denoted by identical superscript letters. Progressive disease indicates documented tumor recurrence or progression without confirmed malignancy-related death. Dates are reported using international formatting (yyyy-mm-dd). PTLD, post-transplant lymphoproliferative disorder; EBV-PTSMT, Epstein-Barr virus-associated post-transplant smooth muscle tumor; BCC, basal cell carcinoma; SCC, squamous cell carcinoma; SCLC, small cell lung cancer; CIS, carcinoma in situ; GC, gastric cancer; Combined VCA and SOT, skull, scalp, pancreas, kidney; e., extremity; m, male; f, female; Clinical, diagnosis by history or examination; Immuno, immunotherapy, Chemo, chemotherapy, Radio, radiotherapy.

**Table 2:** Summary of the immunologic and immunosuppressive characteristics of the combined cohort.

Author	Type of malignancy	Induction of IS	Maintenance of IS	IS at time of diagnosis	Treatment with ATG or alemtuzumab	EBV status (recipient)	EBV status (donor)
Zaccardelli et al.	PTLD	ATG	Tac., MMF, pred.	Tac., MMF, pred	ATG (2011-10)	IgG+/IgM-	n/a
Conrad et al. ^a^	Lymphoma	ATG	Tac., MMF, pred.	Tac., MMF, pred	ATG (2009-11), alemtuzumab (prior to 2010-04)	negative	positive
Conrad et al. ^a^	EBV-PTSMT	ATG	Tac., MMF, pred	Tac., MMF discontinued, pred. n/a)	ATG (2009-11, initiation of IS), alemtuzumab (timing not specified, prior to 2010-04)	negative	positive
Landin et al.	BCC	ATG	Tac., MMF, pred	Siro., MMF, pred.	ATG (2006, initiation of IS)	n/a	n/a
Özkan et al. ^b^	PTLD	ATG, pred.	Tac., MMF, pred	Tac., MMF, pred. (reduced, but reduction no specified)	ATG (2013-08)	IgG+/IgM-	n/a
Özkan et al. ^b^	SCC	ATG, pred.	Tac., MMF, pred.	Tac., MMF, pred.	ATG (2013-08)	IgG+/IgM-	n/a
Cavadas et al.	PTLD	Alemtuzumab	Tac., MMF, pred	Tac., MMF, pred. not specified	Alemtuzumab (2011-07)	positive	negative
Jozaghi et al.	BCC	ATG, methylpred.,	Tac., MMF, pred	Tac., MMF, pred	ATG (2015-05)	positive	positive
Kanitakis et al. ^c^	BCC	ATG, tac., MMF, pred.	Siro. (switch to Tac. 2014-11), MMF, pred.	Siro., MMF, pred.	ATG (2005-11)	n/a	n/a
Morelon et al. ^c^	SCLC	ATG, tac., MMF, pred.	Siro. (switch to Tac. 2014-11), MMF, pred.	Tac., MMF, pred.	ATG (2005-11)	n/a	n/a
Kanitakis et al. ^c^	HPV+ CIS	ATG, tac., MMF, pred.	Siro. (switch to Tac. 2014-11), MMF, pred.	Siro., MMF, prednisone	ATG (2005-11)	n/a	n/a
Kermer et al.	SCC	ATG	Tac., MMF, pred.	Tac., MMF, pred.	ATG	n/a	n/a
Hautz et al.	GC	Alemtuzumab	Tac., MMF	(cessation of IS after VCA-explantation)	Alemtuzumab (2009-07, initiation of IS)	n/a	n/a
Hautz et al. ^d^	BCC	ATG	Tac., MMF, steroids	n/a	ATG (2000-03)	n/a	n/a
Hautz et al. ^d^	SCC	ATG	Tac., MMF, steroids	n/a	ATG (2000-03)	n/a	n/a
Kollar et al.	HCC	n/a	Tac., MMF, pred.	Tac., MMF	n/a	n/a	n/a
Cipriani et al.	PTLD	Alemtuzumab	Tac.	Tac.	Alemtuzumab (2005-02)	n/a	n/a
Kaufman et al.	PTLD	Alemtuzumab	Tac., MMF	Tac., MMF	Alemtuzumab (2006)	positive	positive
Registry case 1	PTLD	n/a	Tac., mycophenolate (MMF/mycophenolic acid)	no longitudinal description	n/a	negative	n/a
Registry case 2	SCC	n/a	Tac., MMF, siro., steroids (pred., methylpred.)	no longitudinal description	n/a	positive	n/a
Registry case 2	BCC	n/a	Tac., MMF, siro., steroids (pred., methylpred.)	no longitudinal description	n/a	positive	n/a
Registry case 2	Prostate Cancer	n/a	Tac., MMF, siro., steroids (pred., methylpred.)	no longitudinal description	n/a	positive	n/a
Registry case 3	PTLD	n/a	Tac., AZA, evero.	no longitudinal description	n/a	negative	n/a

Identical patients with multiple malignancies are denoted by identical superscript letters. The order of patients is identical to **Table 1**. Author and type of malignancy were included to enhance clarity. Dates are reported using international formatting (yyyy-mm-dd). IS, Immunosuppression; EBV, Epstein-Barr virus; PTLD, post-transplant lymphoproliferative disorder; EBV-PTSMT, Epstein-Barr virus-associated post-transplant smooth muscle tumor; BCC, basal cell carcinoma; SCC, squamous cell carcinoma; SCLC, small cell lung cancer; CIS, carcinoma in situ; GC, gastric cancer; ATG, anti-thymocyte globulin; tac., tacrolimus; MMF, mycophenolate mofetil; pred., prednisolone; IgG, immunoglobulin G; IgM, immunoglobulin M; methylpred., methylprednisolone; siro., sirolimus; AZA, azathioprine; evero., everolimus.

### 2.1 Systematic search

We searched PubMed/MEDLINE, EMBASE, and Web of Science on March 2, 2026, for articles on malignancies following vascularized composite allotransplantation (VCA). The search combined VCA-related terms (e.g., “vascularized composite allotransplantation”, “VCA”) and malignancy-related terms (e.g., “tumor”, “PTLD”) using the Boolean operator “AND”. MeSH (Medical Subject Headings) terms and synonyms were included to ensure comprehensive retrieval (**Supplementary Table 1**).

Additionally, we identified eligible studies by manually screening the reference lists of all included articles. Eligible studies reported malignancies occurring after VCA and were published in English with full-text availability. Studies without original data (e.g., registry reports, reviews, meta-analyses), as well as animal and *in vitro* studies, were excluded.

Two reviewers (T.S. and D.D.) independently conducted title and abstract screening followed by a full-text review of eligible studies. Screening and data extraction were performed using Covidence systematic review software (Veritas Health Innovation, Melbourne, Australia, www.covidence.org, RRID (Research Resource Identifier): SCR_016484. Any disagreements were resolved through discussion with a senior reviewer (L.K.).

### 2.2 Quality assessment

The methodological quality of included articles was assessed using the Joanna Briggs Institute (JBI) Critical Appraisal Checklist for case reports and case series (25, 26).

### 2.3 Data extraction

Using a blinded, dual review process (T.S. and D.D.), the following variables were extracted: Study characteristics (first author, year of publication, title, Digital Object Identifier (DOI), country, transplantation center), transplant related variables (type and indication of VCA), donor and recipient characteristics (age and sex) and malignancy-related variables (tumor type, time to diagnosis, detection method, localization, treatment and outcomes). Additional outcomes included graft involvement, patient survival, cause and timing of death, and overall follow-up duration.

## 3 Results

The results are presented as a systematic review (4.1), followed by a combined analysis integrating registry and literature-derived cases (4.2). A detailed description of the three OPTN cases is provided in **Supplementary Text 1**.

### 3.1 Systematic review

**Figure 1:**
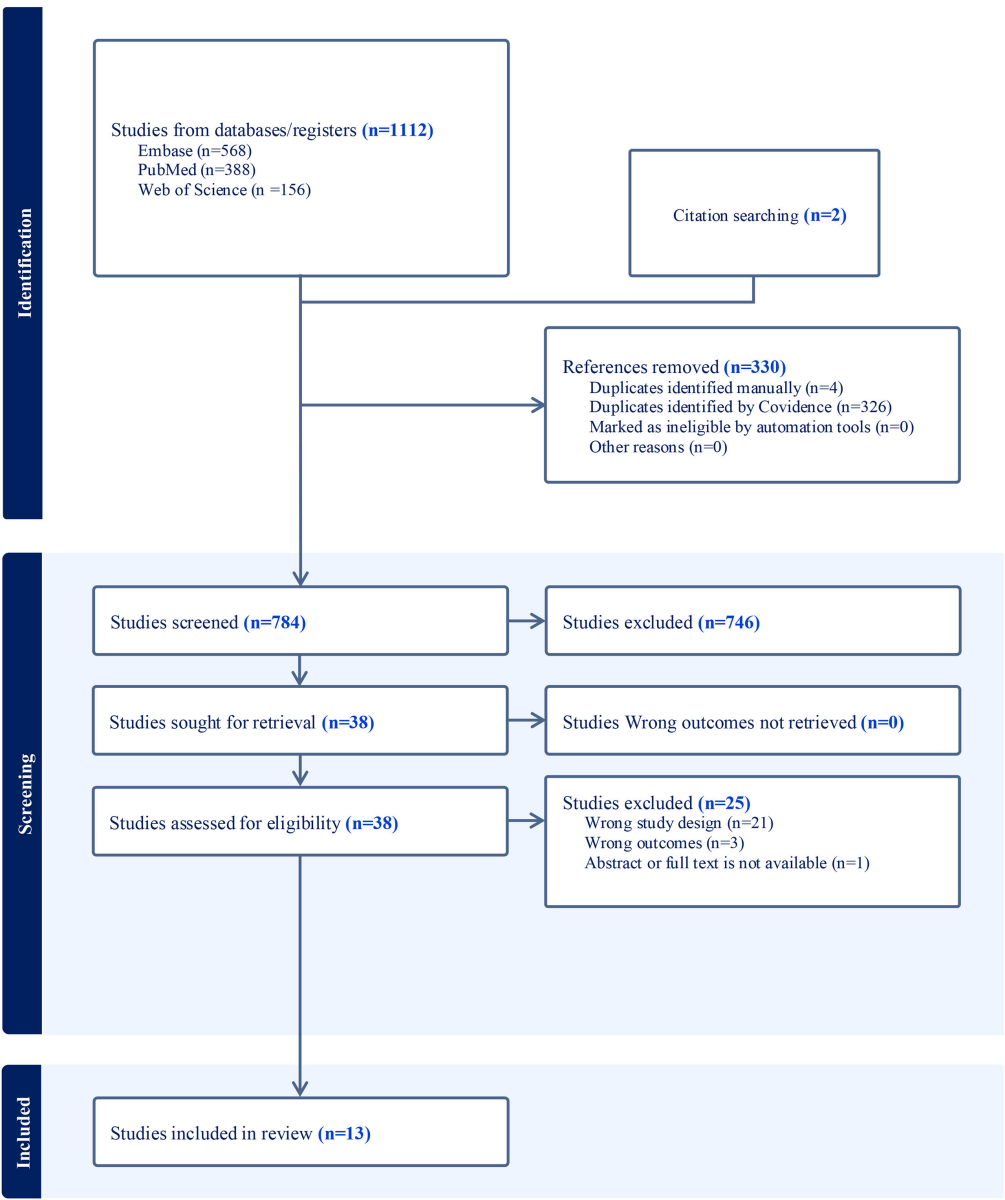
PRISMA flow diagram.

Our search identified 1,112 articles, with eleven meeting the inclusion criteria. Two studies were identified by the manual assessment of reference screening, resulting in 13 included articles. All were case reports (62%; n=8) or case series (38%; n=5), published between 2010 and 2024, predominantly from the US (31%; n=4), Austria (15%; n=2), France (15%; n=2) and Spain (15%; n=2) (**Figure 1**).

#### 3.1.1 Patient demographics of systematic review cases

A total of 13 (100%) VCA recipients were included. The recipient cohort was predominantly male (85%; n=11). Donor sex was female in 23% (n=3), unreported in 62% (n=8) and male in 15% (n=2). Recipient age ranged from 38 to 65 (mean 46 ± 13 years), and donor age from 23 to 46 (mean 33 ± 10) years (**Table 3**).

**Table 3:** Characteristics of post-VCA malignancies identified through systematic review.

Characteristic	Total (13 patients, 18 malignancies)
Patient Age	46 ± 13
Patient Gender [m]	85% (n=11)
Indication of VCA	
*Trauma*	54% (n=7)
*Sepsis, infarction, surgery, radiotherapy*	Each 8% (n=1)
*Not reported*	15% (n=2)
Type of VCA	
*Face*	31% (n=4)
*Unilateral upper extremity*	15% (n=2)
*Bilateral upper extremity*	23% (n=3)
*Others*	31% (n=4)
Follow-up-Time [months]	96 (IQR: 11-125) range: 10-228
Death	54% (n=7)
*Death by malignancy*	39% (n=5)
Time until death [months]	74 ± 58
Type of malignancy	
*Lymphoproliferative disorder*	39% (n=7)
*NMSC*	39% (n=7)
*Solid malignancy*	17% (n=4)
*HPV+ CIS*	6% (n=1)
Time until occurrence [months]	18.5 (IQR: 12-70); range: 5-125
Modality of initial detection	
*Clinical anamnesis/examination*	39% (n=7)
*Imaging*	17% (n=3)
*Routine blood draw*	6% (n=1)
*Not reported*	39% (n=7)
Effect on graft function	22% (n=4)
*Graft loss*	11% (n=2)
*Rejection*	6% (n=1)
* Retransplantation contraindicated*	6% (n=1)
* Change of immunosuppressive regimen*	28% (n=5)
Outcome	
*Remission*	56% (n=10)
*Stable*	11% (n=2)
*Progressive disease*	6% (n=1)
*Death*	28% (n=5)

Categorical variables are presented as absolute numbers and percentages. Continuous variables are presented as mean ± SD. Follow-Up Time and Time until occurrence are presented as median (IQR:Q1-Q3) and range. All values are rounded to the nearest whole number. VCA, vascularized composite allograft; m, male; NMSC, non-melanoma skin cancer, CIS, carcinoma in situ. Treatment modalities are described in the main text and **Table 1**. Percentages may not sum to 100% due to rounding.

The most common indication of VCA was trauma (54%; n=7). Other indications included sepsis-related limb loss, radiotherapy complication, tumor resection, and infarction (each 8%; n=1). In 15% (n=2) indication was not reported.

Facial VCA was most frequent (31%; n=4), followed by bilateral (23%; n=3) and unilateral (15%; n=2) upper extremity transplants. Single cases (each 8%; n=1) included abdominal wall, tongue, bilateral lower extremity and combined multi-organ transplantation (scalp, skull, pancreas, and kidney). Median follow-up was 96 [interquartile range (IQR): 11-125] (range 10-228) months. Overall mortality was 54% (n=7), with 39% (n=5) of deaths attributable to malignancy and 16% (n=2) to cardiovascular compromise. Time from VCA to death was 74 ± 58 (range 11-137) months.

#### 3.1.2 Malignancies following VCA in systematic review cases

A total of 18 (100%) malignancies were identified. Median time to occurrence was 18.5 (IQR: 12-70) months, ranging from 5 to 125 months.

In one patient, gastric cancer (GC) developed one year after VCA explantation and cessation of immunotherapy (9). Most commonly, lymphoproliferative disorders (39%; n=7) and NMSC (39%; n=7), specifically basal cell carcinoma (BCC) (22%; n=4), followed by SCC (11%; n=2) were diagnosed. One SCC represented a recurrence of the primary tumor (18). Single instance malignancies (6%; n=1) included hepatocellular carcinoma (HCC), small cell lung carcinoma (SCLC), GC and human papillomavirus (HPV)-positive cervical carcinoma *in situ* (9, 17, 23, 27, 28).

Initial detection was most commonly based on clinical symptoms (39%; n=7), imaging (17%; n=3) and routine blood work (6%; n=1).

Importantly, no malignancy occurred on the graft site.

Treatment modalities differed between tumors. Surgery was performed in 39% (n=7) of malignancies, while immunochemotherapy was applied in 11% (n=2) of cases and sole chemotherapy in 11% (n=2). A chemotherapy/radiation and local ablation was utilized in one (6%) case each. In 17% (n=3) of cases, there was no malignancy-specific treatment reported. Modification of immunosuppression was reported in 28% (5/18) of malignancies (16, 23, 29–31).

No effect on graft function was reported in 78% (n=14) of malignancies, whereas graft explantation was required in 11% (n=2) of malignancies. One malignancy (6%) was associated with acute rejection and in one patient (6%), malignancy deferred retransplantation since it was discovered during evaluation of retransplantation (17).

Remission was achieved in 56% (n=10), and stable disease in 11% (n=2) of malignancies. Malignancy-related mortality occurred in 28% (n=5) of malignancies of the systematic review. Progressive disease was documented in one malignancy (6%), representing SCC recurrence in a patient who subsequently died from suspected pulmonary embolism (18).

### 3.2 Combined analysis and quality assessment

To provide a comprehensive overview of post-VCA malignancies, we conducted a combined analysis of registry cases and literature-derived cases, which is displayed in **Supplementary Table 2**. After cross-checking the records, we identified one potential overlap between registry-derived case 1 and the literature-derived cases presented by Kaufman et al.; this case was retained in **Tables 1**, **2** for transparency but counted only once when conducting the combined analysis to avoid duplication (31). Accordingly, the combined cohort comprised 15 patients with 22 malignancies, including two additional, non-overlapping OPTN-registry cases.

Methodological quality was assessed using the JBI Critical Appraisal Checklist for case reports and case series. Among the eight included case reports, five studies achieved a score of 100%, two studies achieved 87.5% (Kaufman et al., Kollar et al.), and one study achieved 62.5% (Kermer et al.), primarily due to incomplete reporting of clinical condition and adverse events, resulting in a mean score of 92.2%. Among the five included case series, two studies achieved a score of 100% (Özkan et al., Hautz et al.), while three studies (Conrad et al., Kanitakis et al., Cipriani et al.) scored 77.8%, resulting in a mean score of 86.7%. Overall, the included studies demonstrated moderate to high methodological quality. (**Supplementary Tables 3**, **4**).

## 4 Discussion

In the last decades, VCA has emerged as a treatment option in reconstructive surgery. Yet the long-term oncologic risk in these patients has not been studied extensively. Post-transplant malignancies represent a major complication in SOT and their potential role in VCA remains insufficiently described in the literature. Unless otherwise specified, percentages in the discussion refer to the combined cohort (n=15 patients, n=22 malignancies).

### 4.1 Oncologic risk in comparison to SOT

Any comparison between post-VCA and post-SOT malignancy rates must be prefaced by a critical caveat. The median follow-up in our cohort was 38 (IQR: 12-121) months, which is substantially shorter than the observation periods from which SOT malignancy incidence figures are derived, which routinely extend beyond 20 years (20). This discrepancy alone would be expected to yield lower apparent rates in VCA recipients, irrespective of any genuine biological differences. All subsequent comparisons should be interpreted in light of this fundamental methodological asymmetry.

Beyond follow-up duration, the two populations differ substantially in baseline characteristics, which further complicates any direct comparison of malignancy risk. In a systematic review with 100 patients evaluating immunologic outcome after VCA, Geoghegan et al. reported a mean patient age of 37 years, with trauma (50%) and burns (15%) as the most common indications for VCA (32). In contrast, in a population-based 30-year cohort study in Finland, Friman et al. reported a median patient age of 45 years in SOT recipients, whose primary indication is end-stage organ failure (20, 33). Both advanced age and pre-existing comorbidities (such as chronic viral hepatitis and metabolic disease) are associated with an increased risk of malignancies in SOT recipients (34–36). Thus, the greater age and comorbidity burden typically observed in SOT populations could represent an additional factor that distinguishes these cohorts from VCA recipients and limits direct comparability. Notably, the mean age in our cohort (46 years) was higher than in previously reported VCA populations, suggesting that age may also play an important role in the oncologic risk profile of VCA recipients.

Perhaps most critically, the absence of a reliable denominator and publication bias inherent to VCA case reporting preclude calculation of a true malignancy incidence. Similarly, the limited number of cases per VCA subtype precludes any meaningful comparison of malignancy incidence between different types of VCA. Therefore, any comparison with SOT cohorts remains descriptive rather than epidemiological.

### 4.2 Malignancy types compared to SOT

The malignancy spectrum in our VCA cohort mirrors that in large SOT cohorts, with NMSC (41%) and lymphoproliferative malignancies (36%) dominating the spectrum (19, 20). This similarity may reflect the effect of continuous immunosuppression, particularly impaired tumor immune surveillance and increased susceptibility to oncogenic viral infections (36). In our VCA cohort, solid malignancies comprised only 18% of malignancies, substantially fewer than the 25–30% observed in large SOT cohorts where kidney (7–11%) and lung cancer (9–13%) rank among the most frequent non-cutaneous malignancies (19, 20). This difference likely reflects the distinct indications for VCA (predominantly trauma/burns) versus SOT (chronic organ failure), as the latter are associated with established risk factors for solid organ tumors such as dialysis-related renal cell carcinoma and smoking-associated lung cancer (32, 37, 38).

### 4.3 Impact of immunosuppressive burden and Epstein-Barr virus status

The use of anti-thymocyte globulin (ATG) and alemtuzumab is associated with an increased risk of PTLD (39). In our cohort, all patients for whom induction data was reported received induction of immunosuppression with ATG or alemtuzumab, while induction regimen was not documented for a small number of cases (**Table 2**). Lymphoproliferative disorders were diagnosed almost exclusively in patients who had received one of these T-cell depleting agents, consistent with the hypothesis that lymphodepleting induction may contribute to PTLD risk (40). However, the limited number of patients and the composition of our cohort do not allow for a direct comparison between patients treated with these substances and those who were not, nor can causality be established from a case-series design.

Furthermore, EBV-mismatch, particularly the donor-positive/recipient-negative (D+/R-) constellation, is associated with an increased risk of developing PTLD in SOT (41, 42). Donor EBV status and recipient EBV status was incompletely reported across our cohort and thus preclude systematic evaluation of this risk factor.

Nevertheless, two cases illustrate these considerations. The patient reported by Conrad et al. received ATG for induction and acute rejection episodes were treated with 2 injections of alemtuzumab (30). Additionally, this patient was EBV-seronegative and had received a VCA from an EBV-seropositive donor. In postoperative month 5, he was diagnosed with EBV-positive lymphoma, followed by Epstein-Barr virus-associated post-transplant smooth muscle tumor (EBV-PTSMT) 24 months postoperatively. Conversely, the patient reported from Cavadas et al. received alemtuzumab for induction, was EBV-seropositive and had received a bilateral lower extremity VCA from an EBV-seronegative donor. He developed PTLD 15 months postoperatively (16). These cases may suggest that mechanisms of post-VCA PTLD mirror those suspected in SOT, namely donor-derived primary infection in a seronegative recipient, reactivation of latent EBV in an already-seropositive recipient and immunosuppressive regimens utilizing T-cell depleting agents (39, 41, 43).

### 4.4 Clinical implications

Notably, no malignancy arose at the graft site in any of the included cases, a finding consistent with systemic immunosuppression-related mechanisms as the predominant driver in VCA recipients, rather than local graft-specific factors.

In our cohort, 32% of malignancies were detected by clinical symptoms, 14% by imaging, 5% by routine blood work. Notably, two patients presented with preexisting symptoms. One patient with facial VCA reported having noticed morphologic changes attributable to BCC months before consultation, another reported having noticed night sweats, dyspnea and swelling before a scheduled consultation (28, 29). Malignancy-associated mortality was 33%. These cases indicate that thorough patient education and standardized follow-up protocols may be helpful to mitigate complications of post-VCA malignancies.

Additionally, one SCC recurred fatally, and one HCC arose in the setting of pre-existing cirrhosis, highlighting the potential benefit of pre-transplant oncologic assessment (18, 28). The elective nature of VCA warrants careful patient selection. While evidence is limited, factors such as prior malignancy and higher age may be considered during pre-operative risk stratification. In selected higher-risk patients, conventional reconstructive approaches that do not require lifelong immunosuppression may represent an alternative. Notably, 28% (5/18) literature-derived malignancies were managed with modification of immunosuppression, highlighting its potential role in the management of post-VCA malignancies. Registry cases provided insufficient treatment data for this analysis. Since 41% were NMSC, structured dermatologic screening may be considered as part of routine post-VCA surveillance. Established frameworks in SOT recommend screening intervals ranging from annual examination to every 3 months depending on patient-specific risk (44, 45). Risk-stratification tools such as the Skin and UV Neoplasia Transplant Risk Assessment Calculator (SUNTRAC) have been validated in large SOT cohorts but are not validated in VCA populations (46, 47). The small sample size of our cohort precludes any systematic evaluation of such tools in the VCA setting. Nevertheless, the proportion of NMSC in this cohort highlights the importance of establishing and validating such guidelines specifically in the context of VCA.

## 5 Limitations

While our analysis is limited, it nevertheless provides the most comprehensive overview of post-VCA malignancies to date. First, our findings are exclusively derived from case reports (62%; n=8) and small case series (38%; n=5), thereby potentially introducing publication bias towards severe or unusual malignancies, while underrepresenting milder or asymptomatic cases. In addition, the small sample size and variable follow-up durations (7-228 months in the combined cohort), which are considerably shorter than those reported in the SOT literature, may underestimate late-onset malignancies and limit comparability with SOT cohorts. Similarly, the three registry cases suffer from voluntary reporting bias and incomplete long-term follow-up. For example, the type of VCA is unknown for one patient, two patients were lost to follow-up and the most recent follow-up data for one case date back to 2008. Additionally, registry-derived cases may overlap with previously published cases. Notably, one registry case shared clinical features consistent with the case reported by Kaufman et al., raising the possibility of case duplication. Therefore, this case was counted only once in the combined analysis. (**Tables 1**, **2**) Moreover, due to the absence of a reliable denominator, the true incidence of malignancies after VCA cannot be determined with absolute certainty. Differences in surveillance strategies and diagnostic approaches across centers may have influenced the timing and detection of malignancies.

Additionally, two cases warrant separate consideration. In one case, gastric cancer was diagnosed after graft explantation and cessation of immunosuppression (9). Thus, this case may represent an incidental occurrence rather than a direct consequence of VCA and the associated immunosuppressive burden per se. Additionally, one patient underwent VCA in the setting of preexisting, non-VCA kidney-pancreas transplantation that remained stable for over 20 years (48). Both cases were retained rather than excluded, as our aim was to provide a complete and transparent account of all malignancies identified through our systematic search. Given that VCA remains a relatively young and experimental field, with only a limited number of cases reported worldwide, the exclusion of atypical cases would further reduce an already small evidence base and risk introducing selection bias into a field where comprehensive reporting is still necessary to characterize the true oncologic risk profile. This inclusive approach introduces heterogeneity into the cohort and should be considered when interpreting the reported malignancy spectrum.

Despite these limitations, our findings suggest that the spectrum of post-VCA malignancies mirrors that observed in large SOT registries. Furthermore, our analysis offers initial insights that may inform clinical strategies, such as enhanced dermatologic surveillance given the 41% NMSC prevalence and cautious patient selection for this elective intervention.

## 6 Conclusion

Post-VCA malignancies are a clinically relevant late complication mirroring the oncologic burden seen in solid organ transplantation. NMSC and lymphoproliferative disorders accounted for 41% and 36% of malignancies, a pattern consistent with that observed in chronically immunosuppressed SOT populations, while we found solid organ malignancies to occur less frequently following VCA in comparison to SOT. With a malignancy-associated mortality of 33%, it appears to be prudent to discuss oncologic risk in pre-transplant counseling and patient selection. Dermatologic screening and EBV surveillance represent practical measures that may facilitate early detection. Notably, two patients self-reported symptoms before their scheduled follow-up, suggesting the importance of patient education to facilitate early detection of post-VCA malignancies. As the field of VCA matures, multicenter collaboration and long-term surveillance registries are essential to develop standardized oncologic protocols for the safe practice of VCA.

## Data Availability

The raw data supporting the conclusions of this article will be made available by the authors, without undue reservation.

## Supplementary material

Supplementary Text 1 Description of OPTN registry cases.

Supplementary Table 1 Database search strings and results.

Supplementary Table 2 Characteristics of post-VCA malignancies identified through systematic review and registry analysis. Categorical variables are presented as absolute numbers and percentages. Continuous variables are presented as mean ± SD. Follow-Up Time and Time until occurrence are presented as median (IQR:Q1–Q3) and range. All values are rounded to the nearest whole number. VCA, vascularized composite allograft; m, male; NMSC, non-melanoma skin cancer; CIS, carcinoma in situ. Treatment modalities are described in the main text and **Table 1**. Percentages may not sum to 100% due to rounding.

Supplementary Table 3 Quality assessment using the Joanna Briggs institute (JBI) critical appraisal checklist for case reports. Q1-Q8 items were adapted from the JBI Critical Appraisal Checklist for case reports. Q1, Were patient’s demographic characteristics clearly described? Q2, Was the patient’s history clearly described and presented as a timeline? Q3, Was the current clinical condition of the patient on presentation clearly described? Q4, Were diagnostic tests or assessment methods and the results clearly described? Q5, Was the intervention(s) or treatment procedure(s) clearly described? Q6, Was the post-intervention clinical condition clearly described? Q7, Were adverse events (harms) or unanticipated events identified and described? Q8, Does the case report provide takeaway lessons? Score [%], portion of applicable items answered “yes”, expressed as percentage. 1, yes 0, no.

Supplementary Table 4 Quality assessment using the Joanna Briggs institute (JBI) critical appraisal checklist for case series. Q1–Q10 items were adapted from the JBI Critical Appraisal Checklist for case series. Q1, Were there clear criteria for inclusion in the case series? Q2, Was the condition measured in a standard, reliable way for all participants included in the case series? Q3, Were valid methods used for identification of the condition for all participants included in the case series? Q4, Did the case series have consecutive inclusion of participants? Q5, Did the case series have complete inclusion of participants? Q6, Was there clear reporting of the demographics of the participants in the study? Q7, Was there clear reporting of clinical information of the participants? Q8, Were the outcomes or follow-up results of cases clearly reported? Q9, Was there clear reporting of the presenting site(s)/clinic(s) demographic information? Q10, Was statistical analysis appropriate? Score [%], portion of applicable items answered “yes”, expressed as percentage. 1, yes 0, no U, unclear NA, not applicable.
