# Supplementary material for: Malignancies after vascularized composite allotransplantation
Source: Front Immunol. 2026 Jul 28;17:1905254. doi: 10.3389/fimmu.2026.1905254 (PMC13457038; doi:10.3389/fimmu.2026.1905254)
Supplement: Supplementary file 1 [file SupplementaryFile1.pdf]

12. Supplemental Material

Supplemental Text 1: Description of OPTN Registry Cases

Case 1: An EBV-seronegative 54-year-old male underwent unilateral upper extremity VCA following traumatic partial hand amputation (November 2006). PTLD was diagnosed 23 months post-VCA (October 2008). The patient was alive at last follow-up (November 2008).

Case 2: An EBV-seropositive 56-year-old male patient underwent unilateral upper extremity VCA following traumatic elbow disarticulation (February 2012). Basal cell carcinoma (BCC) was diagnosed at 50 months post-VCA, prostate cancer at 64 months post-VCA and squamous cell carcinoma (SCC) at 74 months post-VCA. The patient was alive 74 months post-VCA but subsequently lost to follow-up (September 2019).

Case 3: An EBV-seronegative 31-year-old female patient received VCA after trauma (February 2019; type not specified). PTLD was diagnosed at an unspecified point in time after transplantation. 6 months post-VCA she remained alive, then lost to follow-up (September 2019).

Supplemental Table 1: Database Search Strings and Results

PubMed/MEDLINE (388)

("Vascularized Composite Allotransplantation"[MeSH Terms] OR "Composite Tissue Allografts"[MeSH Terms] OR "Face Transplant*"[All Fields] OR "Facial Transplant*"[All Fields] OR "Vascularized Composite Allotransplantation*"[All Fields] OR "Vascularized Composite Allograft*"[All Fields] OR "Vascularized Composite Tissue Transplantation"[All Fields] OR "Vascularized Allogeneic Tissue"[All Fields] OR "Vascularized Allograft*"[All Fields] OR "Composite Tissue Allotransplant*"[All Fields] OR "Composite Tissue Allograft*"[All Fields] OR "Composite Tissue Transplant*"[All Fields] OR "Hand Transplant*"[All Fields] OR "Limb Transplant*"[All Fields] OR "Upper extremity transplant*"[All Fields] OR "Lower extremity transplant*"[All Fields] OR "Abdominal wall transplant*"[All Fields] OR "Penis Transplant*"[All Fields] OR "Penile Transplant*"[All Fields] OR"Scalp Transplant*"[All Fields] OR "Larynx Transplant*"[All Fields] OR "Knee Transplant*"[All Fields])

AND

("Neoplasms"[MeSH Terms] OR "Skin Neoplasms"[MeSH Terms] OR "Sarcoma, Kaposi"[MeSH Terms] OR "Lymphoma"[MeSH Terms] OR "Lymphoproliferative Disorders"[MeSH Terms] OR "Carcinoma, Squamous Cell"[MeSH Terms] OR "Carcinoma, Basal Cell"[MeSH Terms] OR "Uterine Cervical Neoplasms"[MeSH Terms] OR "Malignan*"[All Fields] OR "Tumor*"[All Fields] OR "Cancer*"[All Fields] OR"Post-transplant lymphoproliferative disorder*"[All Fields] OR "PTLD*"[All Fields])

EMBASE (568)

('exp vascularized composite allotransplantation/' OR 'exp composite graft/' OR 'Face Transplant*' OR 'Facial Transplant*' OR 'Vascularized Composite Allotransplantation*' OR 'Vascularized Composite Allograft*' OR 'Vascularized Composite Tissue Transplantation' OR 'Vascularized Allogeneic Tissue' OR 'Vascularized Allograft*' OR 'Composite Tissue Allotransplant*' OR 'Composite Tissue Allograft*' OR 'Composite Tissue Transplant*' OR 'Hand Transplant*' OR 'Limb Transplant*' OR 'Upper extremity transplant*' OR 'Lower extremity transplant*' OR 'Abdominal wall transplant*' OR 'Penis Transplant*' OR 'Penile Transplant*' OR 'Scalp Transplant*' OR 'Larynx Transplant*')

AND

('exp Neoplasm/' OR 'exp Skin Neoplasm/' OR 'exp Sarcoma, Kaposi/' OR 'exp Lymphoma/' OR 'exp Lymphoproliferative Disorders/' OR 'exp Carcinoma, Squamous Cell/' OR 'exp Carcinoma, Basal Cell/' OR 'exp Uterine Cervical Neoplasms/' OR 'Malignan*' OR 'Tumor*' OR 'Cancer*' OR 'Post-transplant lymphoproliferative disorder*' OR 'PTLD*')

Web of Science (156)

TS=("Vascularized Composite Allotransplantation" OR "Composite Tissue Allografts" OR "Face Transplant*" OR "Facial Transplant*" OR "Vascularized Composite Allotransplantation*" OR "Vascularized Composite Allograft*" OR "Vascularized Composite Tissue Transplantation" OR "Vascularized Allogeneic Tissue" OR "Vascularized Allograft*" OR "Composite Tissue Allotransplant*" OR "Composite Tissue Allograft*" OR "Composite Tissue Transplant*" OR "Hand Transplant*" OR "Limb Transplant*" OR "Upper extremity transplant*" OR "Lower extremity transplant*" OR "Abdominal wall transplant*" OR "Penis Transplant*" OR "Penile Transplant*" OR "Scalp Transplant*" OR "Larynx Transplant*" OR "Knee Transplant*")

AND

TS=("Neoplasms" OR "Skin Neoplasms" OR "Sarcoma, Kaposi" OR "Lymphoma" OR "Lymphoproliferative Disorders" OR "Carcinoma, Squamous Cell" OR "Carcinoma, Basal Cell" OR "Uterine Cervical Neoplasms" OR "Malignan*" OR "Tumor*" OR "Cancer*" OR "Post-transplant lymphoproliferative disorder*" OR "PTLD*")

Supplemental Table 2: Characteristics of post-VCA malignancies identified through systematic review and registry analysis

Categorical variables are presented as absolute numbers and percentages. Continuous variables are presented as mean ± SD. Follow-Up Time and Time until occurrence are presented as median (IQR:Q1-Q3) and range. All values are rounded to the nearest whole number. Abbreviations: VCA = vascularized composite allograft; m = male; NMSC = non-melanoma skin cancer; CIS = carcinoma in situ. Treatment modalities are described in the main text and Table 2. Percentages may not sum to 100% due to rounding.

**Characteristic**	**Total (15 patients, 22 malignancies)**
Patient Age	46±14
Patient Gender [m]	80% (n=12)
Indication of VCA	
Trauma	53% (n=8)
Sepsis, infarction, surgery, radiotherapy	Each 7% (n=1)
Not reported	20% (n=3)
Type of VCA	
Face	27% (n=4)
Unilateral upper extremity	20% (n=3)
Bilateral upper extremity	20% (n=3)
Others	27% (n=4)
Not reported	7% (n=1)
Follow-Up-Time [months]	38 (IQR: 12-121) range: 7-228
Death	47% (n=7)
Death by malignancy	33% (n=5)
Time until death [months]	74±58
Type of malignancy	
Lymphoproliferative disorder	36% (n=8)
NMSC	41% (n=9)
Solid malignancy	18% (n=4)
HPV+ CIS	5% (n=1)
Time until occurrence [months]	24 (IQR: 12-72); range: 5-125
Modality of initial detection	
Clinical anamnesis/examination	32% (n=7)
Imaging	14% (n=3)
Routine blood draw	5% (n=1)
Not reported	50% (n=11)
Effect on graft function	18% (n=4)
Graft loss	9% (n=2)
Rejection	5% (n=1)
Retransplantation contraindicated	5% (n=1)
Change of immunosuppressive regimen	23% (n=5)
Outcome	
Remission	45% (n=10)
Stable	9% (n=2)
Progressive disease	5% (n=1)
Death	23% (n=5)
Not reported	18% (n=4)

Supplemental Table 3: Quality assessment using the Joanna Briggs Institute (JBI) Critical Appraisal Checklist for case reports.
Note: Q1-Q8 items were adapted from the JBI Critical Appraisal Checklist for case reports.
Q1: Were patient's demographic characteristics clearly described?
Q2: Was the patient's history clearly described and presented as a timeline?
Q3: Was the current clinical condition of the patient on presentation clearly described?
Q4: Were diagnostic tests or assessment methods and the results clearly described?
Q5: Was the intervention(s) or treatment procedure(s) clearly described?
Q6: Was the post-intervention clinical condition clearly described?
Q7: Were adverse events (harms) or unanticipated events identified and described?
Q8: Does the case report provide takeaway lessons?
Score [%]: portion of applicable items answered "yes", expressed as percentage.
1 = yes
0 = no.

**Study**	**Q1**	**Q2**	**Q3**	**Q4**	**Q5**	**Q6**	**Q7**	**Q8**	**Score [%]**
Zaccardelli et al.	1	1	1	1	1	1	1	1	100
Landin et al.	1	1	1	1	1	1	1	1	100
Cavadas et al.	1	1	1	1	1	1	1	1	100
Jozaghi et al.	1	1	1	1	1	1	1	1	100
Morelon et al.	1	1	1	1	1	1	1	1	100
Kermer et al.	1	1	0	0	1	1	0	1	62.5
Kollar et al.	0	1	1	1	1	1	1	1	87.5
Kaufman et al.	0	1	1	1	1	1	1	1	87.5
Mean									92.2

Supplemental Table 4: Quality assessment using the Joanna Briggs Institute (JBI) Critical Appraisal Checklist for case series.
Note: Q1-Q10 items were adapted from the JBI Critical Appraisal Checklist for case series.
Q1: Were there clear criteria for inclusion in the case series*?*
Q2: Was the condition measured in a standard, reliable way for all participants included in the case series?
Q3: Were valid methods used for identification of the condition for all participants included in the case series?
Q4: Did the case series have consecutive inclusion of participants?
Q5: Did the case series have complete inclusion of participants?
Q6: Was there clear reporting of the demographics of the participants in the study?
Q7: Was there clear reporting of clinical information of the participants?
Q8: Were the outcomes or follow-up results of cases clearly reported?
Q 9: Was there clear reporting of the presenting site(s)/clinic(s) demographic information?
Q 10: Was statistical analysis appropriate?
Score [%]: portion of applicable items answered "yes", expressed as percentage.
1 = yes
0 = no
U = unclear
NA = not applicable.

**Study**	**Q1**	**Q2**	**Q3**	**Q4**	**Q5**	**Q6**	**Q7**	**Q8**	**Q9**	**Q10**	**Score [%]**
Conrad et al.	1	1	1	U	U	1	1	1	1	NA	77.8
Özkan et al.	1	1	1	1	1	1	1	1	1	NA	100
Kanitakis et al.	1	1	1	U	U	1	1	1	1	NA	77.8
Hautz et al.	1	1	1	1	1	1	1	1	1	1	100
Cipriani et al.	1	1	1	U	U	1	1	1	1	NA	77.8
Mean											86.7
